# Structural Discrimination of Robustness in Transcriptional Feedforward Loops for Pattern Formation

**DOI:** 10.1371/journal.pone.0016904

**Published:** 2011-02-14

**Authors:** Guillermo Rodrigo, Santiago F. Elena

**Affiliations:** 1 Instituto de Biología Molecular y Celular de Plantas, Consejo Superior de Investigaciones Científicas - Universidad Politécnica de Valencia, Valencia, Spain; 2 Santa Fe Institute, Santa Fe, New Mexico, United States of America; University of South Florida College of Medicine, United States of America

## Abstract

Signaling pathways are interconnected to regulatory circuits for sensing the environment and expressing the appropriate genetic profile. In particular, gradients of diffusing molecules (morphogens) determine cell fate at a given position, dictating development and spatial organization. The feedforward loop (FFL) circuit is among the simplest genetic architectures able to generate one-stripe patterns by operating as an amplitude detection device, where high output levels are achieved at intermediate input ones. Here, using a heuristic optimization-based approach, we dissected the design space containing all possible topologies and parameter values of the FFL circuits. We explored the ability of being sensitive or adaptive to variations in the critical morphogen level where cell fate is switched. We found four different solutions for precision, corresponding to the four incoherent architectures, but remarkably only one mode for adaptiveness, the incoherent type 4 (I4-FFL). We further carried out a theoretical study to unveil the design principle for such structural discrimination, finding that the synergistic action and cooperative binding on the downstream promoter are instrumental to achieve absolute adaptive responses. Subsequently, we analyzed the robustness of these optimal circuits against perturbations in the kinetic parameters and molecular noise, which has allowed us to depict a scenario where adaptiveness, parameter sensitivity and noise tolerance are different, correlated facets of the robustness of the I4-FFL circuit. Strikingly, we showed a strong correlation between the input (environment-related) and the intrinsic (mutation-related) susceptibilities. Finally, we discussed the evolution of incoherent regulations in terms of multifunctionality and robustness.

## Introduction

Complex organisms have evolved precise spatiotemporal control programs, by transducing the presence of signaling molecules to transcription factors, which lead to development and differentiation [1]–[3]. Within this framework, it is important to address the mechanisms by which cells are able to read a gradient of diffusing molecules (morphogens) to trigger the expression of genes that orchestrate spatial organization. The dissection of the minimal genetic architectures that control cell fate [4] will help to understand how a graded signal is transformed into a discrete sequence of states and how fluctuations are counteracted for a robust and precise development. In that way, the natural occurrence in *Drosophila melanogaster* embryos of different networks based on the feedforward loop (FFL) motif for reading morphogen gradients [2], together with the engineering in *Escherichia coli* of synthetic FFL circuits responding in a non-monotonic manner to a graded signal [5]–[7], suggests that this architecture is particularly suitable for pattern formation.

The FFL motif consists in a three-node network where the input regulates the output and a third element, which also regulates the output. FFLs are broadly found both in prokaryotes and eukaryotes and can be seen into eight different architectures depending on the sign of its regulations [8]. Notably, this particular structure has certain functionalities *per se*. Theoretical and experimental work on the incoherent FFL (I-FFL), mostly based on transcriptional regulations but also enzymatic reactions, has revealed its ability to work as an amplitude (concentration) filter [5]–[7], [9]–[11], to accelerate the output response [8], [12], for signal amplification and fold-change detection [13], [14], and to generate temporal pulses in response to a constant stimulus [8]–[10], [15], [16]. Interestingly, this last attribute can be interpreted in terms of adaptiveness, where after a transient behavior the system returns to the previous state, being the output steady state level independent of the input level [17]–[22].

In the present work, we investigate, by dissecting the design space that contains all possible topological configurations (wiring) and kinetic parameter values, whether a single FFL circuit (a topology with certain parameterization) can accommodate both (*i*) the ability to read a gradient by means of an amplitude detection mechanism and (*ii*) the ability to achieve optimal adaptive response at high output levels. Certainly, the capacity for adaptive responses of living organisms (partial or absolute) is an intriguing question in biology, and previous work, mostly based on metabolic systems (bacterial chemotaxis), has pointed out that optimal adaptiveness is more a consequence of circuit topology than of the fine tuning of kinetic parameters [19]–[22]. Thus, although the different I-FFL configurations can yield *a priori* a palette of functionally analogous devices, they may display different robustness profiles against external perturbations (i.e., structural discrimination of robustness).

## Results

### Optimal FFL circuits for pattern formation

We aimed at designing FFL circuits able of generating one-stripe patterns. For that, we computationally explored the whole designing space (FFL architectures and kinetic parameters). Our mathematical model, simultaneously accounting for transcription and translation processes, contains ten parameters (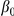, 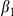, 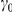, 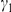, 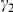, 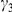, 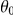, 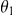, 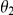, and 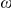) that define the design space (see Materials and Methods). For an efficient exploration, and given that designing space is vast for an exhaustive computation, we adopted a heuristic optimization-based approach. We simplified the spatial diffusion and focused our study on amplitude filtering systems where the output reaches a maximum at intermediate input levels. Analogous results could be obtained for inverse amplitude filters (existence of a minimum). The transfer function is in brief characterized by the input detection amplitude (or bandwidth) and by the output amplitude (ratio between the maximal and basal output concentrations). The shape of this function serves to classify the amplitude filters into those exhibiting precision, i.e., the detection is accomplished at a very accurate position, and those being adaptive, i.e., a wide detection range exists so the stationary output level is insensitive to variations in the input. Certainly, a reliable pattern requires perceptible output amplitude, at least one order of magnitude, to differentiate the two cell fates (ON/OFF). Here, we imposed the condition that the output amplitude must be 100-fold. Nevertheless, there is a clear tradeoff between the bandwidth and the output amplitude, in the sense that a given output amplitude constrains both the maximal and minimal bandwidths that the system can attain. Herein, we considered that the morphogen (the input) interacts at the genetic level by inhibiting post-translationally the regulatory ability of a sensory transcription factor [2]. Similar results can be obtained if the morphogen induces the degradation of that regulator (e.g., proteasome-mediation) or activates it (e.g., phosphorylation). In fact, such a regulatory mode is not very relevant because of the symmetry of the transfer function.

**Figure 1:**
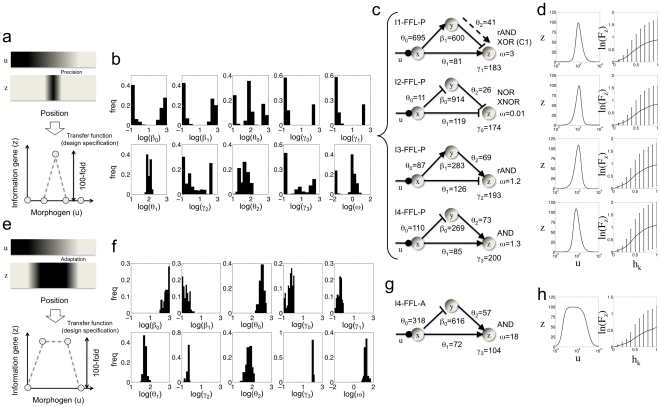
Landscape of FFLs for pattern formation. (a, e) A spatial gradient of an external molecule (morphogen) induces a particular cell fate depending on the position. The simplest pattern consists in a one-stripe composition with two cell fates, triggered by the expression level of one gene. The spatial information can be reduced to construct the transfer function of the system (relation output/input in steady state). (b, f) Histograms for the kinetic parameter values of the model resulting from multiple optimization runs with different initial guesses that explored the design space (see Dataset S1). The ordinates represents the value in logarithmic scale, while the abscises the frequency of each one produced by the heuristic procedure. (c, g) Incoherent FFL architectures together with the average value of the relevant kinetic parameters emerged from the landscape exploration. (d, h) Transfer (left) and sensitivity (right) functions characterizing each circuit (see Materials and Methods). The sensitivity function is calculated at maximal output level.

First, we sought for patterns with maximal precision (Fig. 1a). This entails a transfer function with a narrow bandwidth. In Fig. 1b, we show the histograms for the kinetic parameters that characterize all optimal solutions. Remarkably, these histograms are not dense, indicating that there are few optimal points. In fact, these histograms correspond to four solution modes, which are the four I-FFL architectures with a specific parameterization (Fig. 1c). We denote I1-FFL-P, I2-FFL-P, I3-FFL-P, and I4-FFL-P these four circuits (the P stands for optimized for precision). In Fig. 1d, we plot the transfer, *z*(*u*), and sensitivity, *F_z_*(*h_k_*), functions that characterize the behavior of each circuit (see Materials and Methods). These circuits show no qualitative differences in the two functions, suggesting that the four architectures are equally good at precision. Indeed, these circuits rely on a mechanism based on a tradeoff between the two regulatory branches, which have opposite sign. At high input levels, both activation and repression branches are inactive (state OFF), and at low ones both branches are active, accomplishing the state OFF because repression is dominant. While, at intermediate input levels, the activation branch is active and the repression inactive (state ON). For circuits I1-FFL-P and I3-FFL-P, rAND means that the output gene is expressed in presence of the activator and absence of the repressor. Interestingly, we found that the C1-FFL architecture with a combinatorial logic type XOR (i.e., the activators inhibit each other) and a weak activation from the intermediary gene to the output is also a solution. With the exclusive logic, this is in fact an I1-FFL variant. In addition, circuit I2-FFL-P emerged with either a combinatorial logic type NOR (i.e., the repressors act independently each other) or XNOR (i.e., the repressors inhibit each other) and competitive binding. Moreover, circuit I4-FFL-P emerged with a combinatorial logic type AND (i.e., both activators act synergistically) and independent binding. We did not obtained from the landscape exploration further combinatorial logics for circuits I2-FFL-P and I4-FFL-P, which suggests that such configurations would not be plausible because they would not introduce the required tradeoff between the opposite regulatory branches (this can be shown mathematically).

Second, we sought for patterns with optimal adaptive response in the state ON (Fig. 1e). This entails a transfer function with a *plateau*, which gives definitively a wide bandwidth. In Fig. 1f, we show the histograms for the kinetic parameters that characterize all optimal solutions. Surprisingly, all kinetic parameters are highly constrained by the design specifications, which corresponds to just one solution mode, the I4-FFL architecture with a specific parameterization (Fig. 1g). We denote I4-FFL-A this circuit (here A stands for optimized for adaptation). In Fig. 1h, we plot its transfer and sensitivity functions. As it can be observed, this circuit has a wider bandwidth and presents a lower sensitivity to perturbations in the kinetic parameters at the state ON. The circuit emerged with a combinatorial logic type AND and cooperative binding, whose working principle also relies on the tradeoff between the two regulatory branches. On the light of these numerical results, it could be concluded that the optimal adaptive response (existence of a *plateau*) was structurally encoded by the I4-FFL topology and, in contrast to circuit I4-FFL-P, modulated by a strong binding cooperation (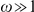) between the two activators.

### Theoretical analysis for patterning and adaptation

Motivated by the numerical results from the heuristic landscape exploration, we performed a theoretical analysis to elucidate the attribute that discriminates the I4-FFL as the central topology with adaptive performance in the state ON. On the one hand, mathematically, the one-stripe pattern condition implies that the output concentration reaches an optimum, which gives the equation 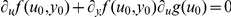 where 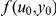 is the production term of *z* and 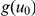 of *y* in the steady state (see Materials and Methods). Certainly, this can be satisfied in case of I-FFL circuits, where the sign of the direct regulatory branch (*x* to *z*) is opposite to that of the indirect branch (*x* to *y* to *z*). This condition only guarantees the presence of an optimum and not a reliable amplitude level. Together, the specification of a desired amplitude level (e.g., 100-fold with respect to the basal state) entails a precise parameterization.

On the other hand, to achieve an absolute adaptive response the output concentration in steady state has to be input-independent regardless the values taken by the kinetic parameters. Only the transient behavior will be affected by such numerical values. For each topology three possibilities exist although for illustrative purposes we will focus on the I1-FFL topology. First, the system will show adaptiveness when 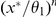 is the dominant term in the denominator of 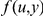, being 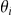 a parameter for binding affinity. In this case, there is a strong activation of x* that saturates the production of *z*, whereas the repression by *y* becomes negligible. Second, when the production of *y* is linear with x* (i.e., 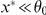 and 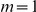) and 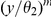 is the dominant term in the denominator of 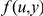. Now, since *y* is proportional to x*, the activation of x* on *z* is counteracted by the strong repression of *y*. Third, when the production of *y* saturates (i.e., 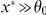) and the cooperative term 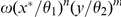 dominates the denominator of 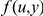. Analogous derivations can be done for the other I-FFL architectures.

**Figure 2:**
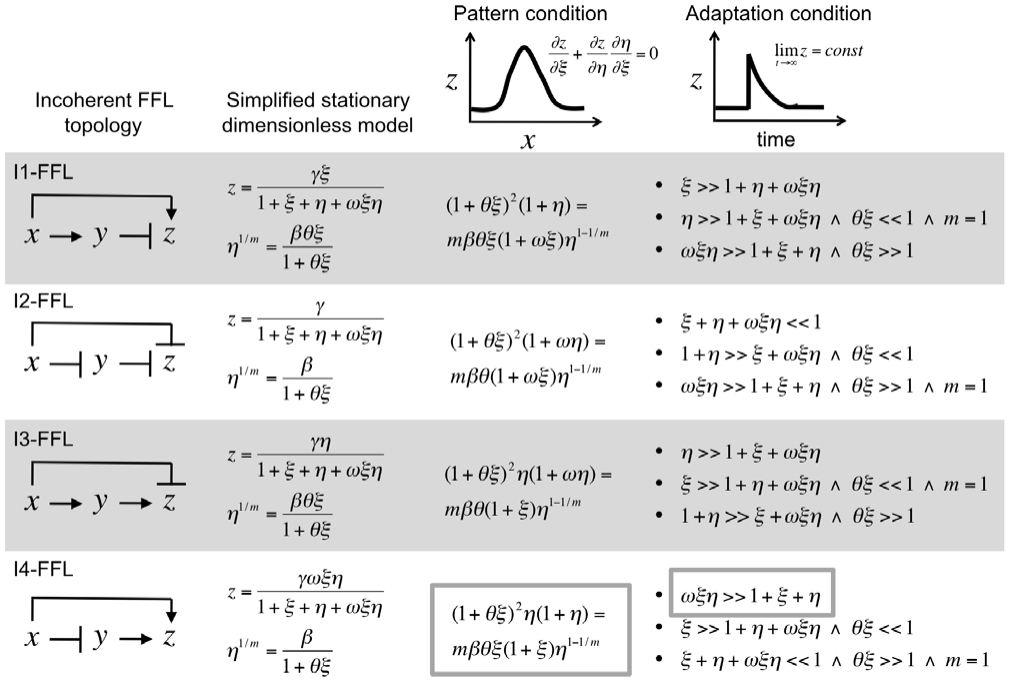
Theoretical analysis of the four I-FFL topologies. The I2-FFL is assumed to have a combinatorial logic type NOR, and the I4-FFL a type AND. We considered a dimensionless model in steady state, where 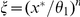 and 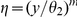, and simplified it to only account for the higher synthesis rate (see Materials and Methods). Moreover, 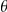, 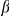 and 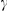 are dimensionless parameters. For each FFL topology, we mathematically derived the condition to achieve pattern formation (i.e., *z* must reach a maximum at intermediate levels of x*) and adaptiveness (i.e., *z* in steady state must be independent of x*). For optimal adaptive response, there are three possible strategies that can be implemented with particular choices of kinetic parameters.

Fig. 2 summarizes all pattern and adaptation conditions for the four I-FFL topologies. The optimality condition, together with a specific amplitude level, imposes a strict relation between some kinetic parameters of the model (mostly those binding-related) and the concentration values of the species. Nevertheless, only for the I4-FFL with a combinatorial logic type AND, that condition is independent of *ω*, which is free to adopt a given value. This fact is a direct consequence of the circuit topology and is instrumental to achieve adaptation at high output levels. By setting a high value of *ω* we can ensure the first adaptive condition for the I4-FFL circuit. In this case, the cooperative term 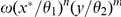 dominates the denominator of 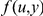, yielding a constant function, and hence the pattern condition is satisfied because 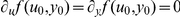.

### Adaptiveness correlates to genetic robustness

**Figure 3:**
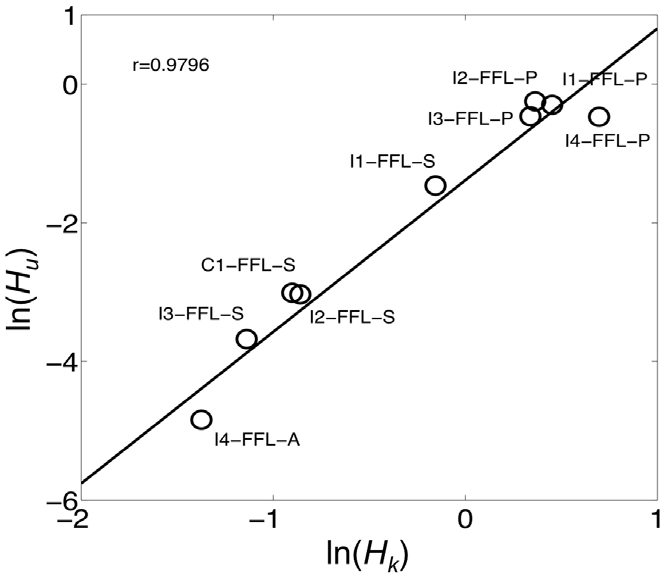
Adaptiveness versus parameter sensitivity. Correlation between the input and intrinsic susceptibilities (*H_u_* and *H_k_* respectively) in natural logarithmic scale (see Materials and Methods), where each circle corresponds to one circuit. For this plot, to calculate *H_k_* we considered the parameters 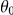, 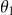, 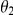, and 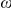. We represent the four circuits optimized for precision (I1-FFL-P, I2-FFL-P, I3-FFL-P and I4-FFL-P), the one optimized for adaptiveness (I4-FFL-A), and four more suboptimal circuits (I1-FFL-S, C1-FFL-S, I2-FFL-S and I3-FFL-S). The corresponding parameter values of all these circuits are provided in Dataset S1. The value of *r* corresponds to the linear correlation coefficient (solid line obtained by linear fit).

We next explored the consequences of adaptiveness in the sense of congruent evolution to genetic robustness [23], [24]. For that, we calculated the susceptibility of the circuit under perturbations in the input level (*H_u_*) and in the kinetic parameters of the model (*H_k_*). We focused our study on circuits operating at the state ON. Here, to calculate the intrinsic susceptibility we just considered variations in the most important parameters, those related to the binding affinities between transcription factors and DNA [2]. Indeed, the amplitude detection mechanism exploits the differences in those binding affinities, and computational studies on the dorso-ventral gradient in *D. melanogaster* embryos have confirmed that these parameters mediate the major control on the expression of target genes [25]. Fig. 3 represents the four circuits optimized for precision (I1-FFL-P, I2-FFL-P, I3-FFL-P, and I4-FFL-P), the one optimized for adaptive response (I4-FFL-A), and four more suboptimal circuits (I1-FFL-S, C1-FFL-S, I2-FFL-S, and I3-FFL-S). Whereas I4-FFL-A achieves optimal adaptive response, it could be argued that the suboptimal circuits exhibit partial adaptation. In logarithmic scale, we show a strong correlation between the input and intrinsic susceptibilities. This fact suggests that the acquired ability of certain biological systems to be robust against mutations that change their kinetic properties is a direct consequence of their ability to respond to environmental perturbations (i.e., environmental robustness). The I-FFL circuit by means of a tuned balance between the two regulatory branches allows counteracting by anticipation any perturbation in the input or in any element upstream the output. However, such a circuit cannot neutralize perturbations in the synthesis rate of the output gene. To do so, the circuit would need to introduce a negative feedback loop (N-FBL) implementing an integral control [18]. In fact, N-FBLs have been shown to provide robustness in transcription [26] and metabolic [22], [27] networks, and its combination with I-FFLs can enhance the robustness performance [22].

### Robustness to noise

**Figure 4:**
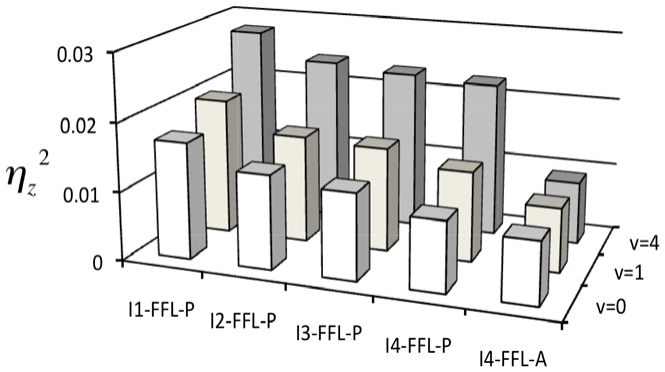
Noise tolerance for optimal designs. Noise in output expression (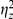) for different FFL circuits due to intrinsic effects and several noise levels at the input; *v* represents the corresponding Fano factor (see Materials and Methods).

In addition to the susceptibility calculations, we carried out a stochastic analysis to study the robustness of the circuits against molecular noise [28]–[31]. We considered an intrinsic source of noise due to the low number of molecules together with a noisy input signal (see Materials and Methods). We performed numerical simulations to calculate the noise level in the output gene at the state ON (Fig. 4) for different noise amplitudes in the input for the optimal circuits (I1-FFL-P, I2-FFL-P, I3-FFL-P, I4-FFL-P, and I4-FFL-A). Essentially, noise in gene expression can be decomposed into three terms, one intrinsic that is Poissonian for genes without self-regulation, another due to propagation effects, and a third extrinsic one accounting for sources common to all species [30]. In our case, we did not consider extrinsic noise, and the propagation term accounts for noise directly resulting from the input (*N_u_*) and noise coming indirectly via the intermediary element (*N_y_*). These terms are proportional to their susceptibilities (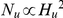, 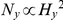). Then we can write the expression 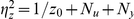 for noise in the output. Circuits with similar transfer functions have similar susceptibilities, however noise tolerance is structure-dependent. Indeed, at the state ON, the concentration of the intermediary element is low for circuits I1-FFL-P and I2-FFL-P because this gene represses the output, whereas it is high for circuits I3-FFL-P and I4-FFL-P as in these cases it activates the output. This fact entails that the term *N_y_* is higher for circuits I1-FFL-P and I2-FFL-P than for circuits I3-FFL-P and I4-FFL-P, since noise is inversely proportional to concentration. As we can observe, noise increases in circuits optimized for precision with randomly fluctuating input signals, whereas circuit I4-FFL-A is highly insensitive to such stochastic events, maintaining a constant Poissonian noise level (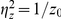). This can be rationalized knowing that 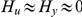 for this circuit. For high input fluctuations (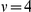), we have 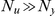 thereby precise circuits show similar noise levels.

## Discussion

The knowledge of the dynamical properties of different fundamental regulatory circuits is crucial to infer the selective pressures that the cell has suffered during its evolution. In fact, although the kinetic parameters are important to determine the dynamical behavior, a circuit topology by itself can determine or constrain the dynamics and provide structural sources of robustness [19]–[21] or noise tolerance [32]. Why a precise regulatory motif is prominent in Nature, whereas a functionally analogous circuit (same behavior but different topology) is less abundant or even not found, remains an intriguing question. Certainly, depending on the biological demands, a particular circuit will be more favorable for the cell. Regulatory circuits based on I-FFLs can operate in a dosage-response manner to generate one-stripe spatial patterns. More complex (multiple-stripe) patterns can be obtained by interplaying several I-FFLs [9]. In fact, the segmentation network of *D. melanogaster* involves several cascades of genes that allow obtaining these banding patterns. For instance, while the *gap* genes form a one-stripe pattern, the downstream elements, such as the pair-rule or segmentation genes, give multiple-stripe patterns [33], [34]. Importantly, this supports the modular organization of the regulatory networks by which complex functions are reached by interconnecting small units. The four I-FFL architectures, with a proper parameterization, can operate with maximal precision having similar input and intrinsic susceptibilities. However, noise at the state ON is eventually higher for circuits I1-FFL-P and I2-FFL-P due to the monochromatic regulatory mode of the sensor, which leads to a repression exerted by the intermediary element. Remarkably, only the I4-FFL topology is able to provide adaptiveness at the state ON (while the four architectures can give an adaptive response at the state OFF).

In a recent work, Cotterell and Sharpe proposed different three-gene topologies, not necessarily FFLs, to produce one-stripe patterns [35]. Using a systematic design procedure, these authors found new structural elements for reading morphogen gradients and controlling developmental genetic units, some of which should still be discovered *in vivo*. Furthermore, the combination of these elements can enlarge the repertoire of circuit topologies and increase the level of robustness. However, unless bistable-like circuits, these topologies were essentially based on I-FFLs. Furthermore, some canonical functional topologies were mislaid, such as the I4-FFL, indeed because the search algorithm used by Cotterell and Sharpe did not account for synergistic actions (e.g., promoters type AND). Herein, our design procedure has resulted more sensitive to study the transcriptional FFLs and has allowed us to refine such general approaches for a comprehensive study. Our model accounts for the intracellular circuit dynamics under certain level of an external signal and without tolerating the diffusion of proteins. In this sense, Cotterell and Sharpe illustrated that protein diffusion resulting in a cell-to-cell communication weakly affects noise tolerance but results into a mechanism that allows tuning the position and bandwidth of the stripe. Interestingly, diffusion affects the bandwidth differently depending on the circuit structure. Therefore, a logical further step concerning adaptiveness would be to study the addition of more regulations over single FFLs, the effect of diffusion and the signaling at the intermediary gene level to obtain a widespread analysis of the different genetic architectures that allow reading gradients and generate one-stripe patterns.

In addition, the I-FFL motif is also found in simple organisms that do not require the formation of spatial patterns (e.g., bacteria or yeast). In this case, the filtering device normally operates at one state, and switch to the other state after environmental changes. According to the Savageau's demand principle [36], the mode of gene regulation should entail a maximization of the usage (binding to DNA) of the transcription factors; otherwise, the regulators are lost during evolution. On the one hand, in circuits based on I1-FFL and I2-FFL topologies operating at the state ON only one regulator is functional, whereas in case of I3-FFL and I4-FFL topologies the state ON requires the function of the two regulators. This relates to the fact that in the I1-FFL and I2-FFL the sensor has a monochromatic regulatory mode, whereas for the I3-FFL and I4-FFL it acts as activator and repressor simultaneously. Hence, it would be expected that circuits operating at the state ON were preferentially based on I3-FFL and I4-FFL and were present within the regulatory map of highly demanded biological functions, such as central metabolism or transcription-translation machinery. On the other hand, only the I1-FFL entails the functionality of the two regulators at the state OFF but for low input levels, since we are considering that the input post-translationally inhibits the sensor. Then, circuits operating at the state OFF would be mostly based on the I1-FFL and would control genes of low demand (e.g., secondary metabolism) or genes that need to be activated in specific situations such as stress responses or during development. Interestingly, I1-FFL architectures are the most abundant ones in bacteria and yeast [8], being reasonable that this abundance is a consequence of the specialization of the I-FFL to operate as time pulse generator and keep the expression of its target genes tightly suppressed in absence of external stimuli.

One open question that arises from our results is if given the properties of robustness associated to I4-FFLs, their abundance as regulatory module could be considered as an exaptation (an spandrel in S. J. Gould usage) that results from selection of larger and more complex network structures or, perhaps, as a direct consequence of selection for increased robustness [37]. In this second case, the consequent relevant question is how robustness mechanisms were selected for. If buffering mechanisms minimize the effect of every possible mutation, they will operate on the mutations created, thus making them invisible to natural selection and hence preventing their spread in the population. A possible solution to this paradox is that mutational robustness is a side effect of selection for mechanisms that buffer environmental perturbations [23], [24]. Our observation that when we imposed selection against adaptiveness the optimal design I4-FFL-A was also robust against parameter perturbations (equivalent to mutational effects on catalytic/binding properties) gives further support to this possibility. Therefore, the recurrent inference of the design principles that confer adaptiveness to organisms would clarify our understanding of the causes of robustness to genetic perturbations and noise.

## Materials and Methods

### Mathematical model

The FFL motif consists in three genes (*x*, *y* and *z*) and it can indeed appear as eight different architectures, four coherent and four incoherent, depending on the nature of the regulations [8]. In addition, we consider an external molecule (*u*) that modulates the active form of *x* (x*) by post-translational inhibition. Our model parameterizes all these architectures following a Hill-like function formalism [38] and reads
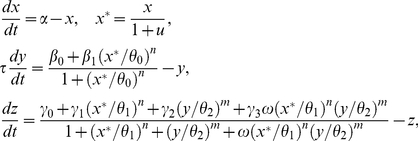
(1)where 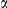 is the synthesis rate of *x* (here 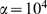), 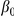 and 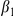 the synthesis rates of *y* from the unregulated and *x*-regulated promoter respectively, and 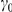, 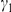, 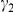, and 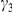 the synthesis rates of *z* from the unregulated, *x*-regulated, *y*-regulated and *x*,*y*-regulated promoter respectively. The regulatory coefficients (bindings protein-DNA) are 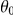, 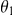, and 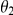, and *n*, *m* are the Hill coefficients. Typically, the active form of a transcription factor to activate/repress the promoter consists of a dimmer, thus for simplicity we fix *n* = *m* = 2 otherwise specified, although it could be straightforward the exploration of higher order aggregations. The parameter 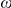 accounts for the potential interaction in the promoter region of *x* and *y*, from competitive (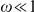) to cooperative binding (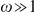). For independent binding, 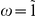 In addition, 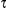 is a dimensionless parameter that accounts for the relative stability of the intermediate protein *y* (here 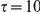), related to the transient behavior but not affecting the stationary value. In case of adaptation, this parameter, which can be viewed as a delay over the expression of *y*, controls the amplitude and duration of the transient response after which the system returns to the original state [8], [13]. This model could be enlarged to account for mRNA dynamics, although for FFL circuits this would not affect the steady state of the system, or slightly modified to account for post-transcriptional regulations, as miRNA-mediated FFLs are recurrently found in mammals [39]. For notation purposes, in steady state we have 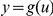 and 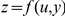, being 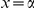; in some cases, we just write 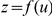.

### Landscape exploration

We followed an optimization scheme based on Monte Carlo Simulated Annealing to efficiently explore the landscape defined by the general FFL model and a scoring function that measures the distance between two functions [40], [41]. We used an exponential cooling scheme, from pure random walk at initial iterations to pure adaptive walk at final ones. Starting from a given parameter set, the algorithm mutated one parameter value at each step, computed the transfer function, 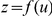, and selected against a target function implementing an amplitude filter (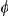). We targeted two different functions, with Lorentzian structure, depending on the bandwidth specification. In case of precision, we targeted 
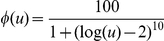
 (narrow bandwidth), whereas in case of adaptation 
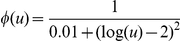
 (wide bandwidth). In both cases, we imposed a maximal amplitude level of *z*_0_ = 100 for *u*_0_ = 100 (in the basal state 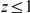). For each parameter we defined a variation range in logarithmic scale (in our particular case, 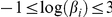, 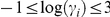, 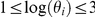, and 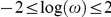). We discretized those ranges into ten values per order of magnitude, giving a design space size of about 10^16^. We run multiple times by starting from different initial conditions with the aim of ensuring a good exploration of the landscape. This heuristic approach allowed us to find the optimal combinations of parameter values. Afterwards, for each circuit we recalculate the particular values of *u*_0_ and *z*_0_, being 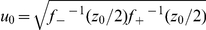 and 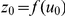.

### Susceptibility and stochasticity

To quantitatively study the robustness of a circuit, we introduced the concept of susceptibility, that is, a measure that relates the change in the output (*z*) from a perturbation in the system (i.e., a change in one variable of the model). Here, we considered two measures: the input susceptibility (*H_u_*), which relates the output level to changes in the input, and the intrinsic susceptibility (*H_k_*), which relates the output level to changes in the kinetic parameters of the model. We also introduced the geometric average output fold-change, 
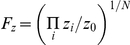
 for *N* perturbations. Then, we indentified the input susceptibility according to 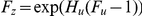, where the variable 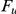 denotes a change in the input of 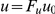 or 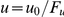. The fit was done by considering 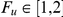. This definition of susceptibility turns out into 
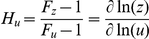
 (the logarithmic gain of the system) for small input perturbations. For the intrinsic susceptibility, we assumed that each parameter (*k*) was a Gaussian distributed random variable with mean its nominal value (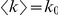) and standard deviation a percentage of it (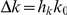). Then, we fit the intrinsic susceptibility to 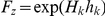, with a range of variation of 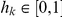.

The stochastic modeling was performed via Langevin formulation [28]–[31]. We assumed that noise in *x* is negligible due to its high synthesis rate. Therefore, noise in x* comes from noise in the input (*u*), whose statistics are 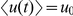 and 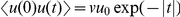, where 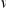 is the Fano factor. We assumed that the diffusion time is of the order of the half-life of protein *x*, which is assumed to be short-lived. For instance, the Bicoid protein diffuses about 0.3 mm^2^/s in *D. melanogaster* embryos of about 100 mm^2^ giving a diffusion time of about 5–6 min [42]. The stochastic model reads
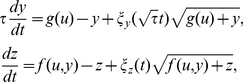
(2)where 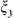 and 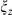 are Wiener processes with statistics 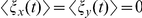 and 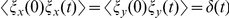. Using perturbation theory (the mean field is deterministic and the perturbation amplitude only depends on the mean field) and Fourier analysis [28]–[31], it is straightforward to show that noise in the output reads 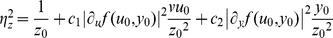, where *y*_0_ and *z*_0_ are the stationary solutions at the state ON, being *c*_1_ and *c*_2_ two constants. By using the concept of susceptibility, with 
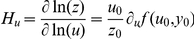
 and 
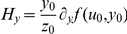
, we can write 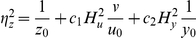.

## Supporting Information

Dataset S1 **Parameter values of 500 independent optimization runs that achieved convergence corresponding to the four solutions for precision and the one for adaptation.** It also contains the parameter values for the optimal circuits I1-FFL-P, I2-FFL-P, I3-FFL-P, I4-FFL-P, and I4-FFL-A.
(XLS)
